# Neuronal Assemblies Evidence Distributed Interactions within a Tactile Discrimination Task in Rats

**DOI:** 10.3389/fncir.2017.00114

**Published:** 2018-01-11

**Authors:** Camila S. Deolindo, Ana C. B. Kunicki, Maria I. da Silva, Fabrício Lima Brasil, Renan C. Moioli

**Affiliations:** Graduate Program in Neuroengineering, Edmond and Lily Safra International Institute of Neuroscience, Santos Dumont Institute, Macaiba, Brazil

**Keywords:** neuronal assemblies, distributed cortical interactions, tactile discrimination, rat, independent component analysis

## Abstract

Accumulating evidence suggests that neural interactions are distributed and relate to animal behavior, but many open questions remain. The neural assembly hypothesis, formulated by Hebb, states that synchronously active single neurons may transiently organize into functional neural circuits—neuronal assemblies (NAs)—and that would constitute the fundamental unit of information processing in the brain. However, the formation, vanishing, and temporal evolution of NAs are not fully understood. In particular, characterizing NAs in multiple brain regions over the course of behavioral tasks is relevant to assess the highly distributed nature of brain processing. In the context of NA characterization, active tactile discrimination tasks with rats are elucidative because they engage several cortical areas in the processing of information that are otherwise masked in passive or anesthetized scenarios. In this work, we investigate the dynamic formation of NAs within and among four different cortical regions in long-range fronto-parieto-occipital networks (primary somatosensory, primary visual, prefrontal, and posterior parietal cortices), simultaneously recorded from seven rats engaged in an active tactile discrimination task. Our results first confirm that task-related neuronal firing rate dynamics in all four regions is significantly modulated. Notably, a support vector machine decoder reveals that neural populations contain more information about the tactile stimulus than the majority of single neurons alone. Then, over the course of the task, we identify the emergence and vanishing of NAs whose participating neurons are shown to contain more information about animal behavior than randomly chosen neurons. Taken together, our results further support the role of multiple and distributed neurons as the functional unit of information processing in the brain (NA hypothesis) and their link to active animal behavior.

## 1. Introduction

Recent years have seen a surge in neuronal *ensemble* recording technology, making it feasible to simultaneously record from multiple brain areas hundreds to thousands of cells in behaving animals (Laubach et al., 1999; Gutierrez et al., 2005; Lebedev and Nicolelis, 2006; Wiest et al., 2007; Long and Carmena, 2011; Aggarwal et al., 2013; Schwarz et al., 2014). Studies have investigated how this massive amount of data relate to the functioning of neural circuits (Stevenson and Kording, 2011) and, ultimately, to animal behavior (Mohammed et al., 2016), but many open questions still remain. Following Hebb's description of the synaptic activity (Hebb, 1949), the neuronal *assembly* (NA) hypothesis propose that subgroups of co-activated cells constitute the fundamental unit of information processing in the brain (Nicolelis et al., 1997; Harris, 2005; Buzsáki, 2010). Importantly, subgroups may encompass distant neurons, distributed in different regions, possibly forming complex neuronal circuits through indirect connections or due to a common input (Buzsáki, 2010). The existence of NAs is widely discussed (Gerstein et al., 1989; Buzsáki, 2006; Picado-Muino et al., 2013; Russo and Durstewitz, 2017), as it correlates with several neurological phenomena (Sakurai, 1998; Fries, 2005; Buehlmann and Deco, 2008; Engel et al., 2013). However, despite the mounting experimental evidence supporting NAs and distributed brain interactions (Bower et al., 2015; Valdez et al., 2015; Carrillo-Reid et al., 2016; Mishra et al., 2016), their experimental verification is still a grand challenge in neuroscience.

The Hebbian theory sought to reconcile single-neuron dynamics to complex psychological phenomena, proposing that: (1) assembly neurons fire synchronously and as a consequence reinforce their synaptic efficiency; (2) one neuron may participate simultaneously in multiple NAs; (3) information coding is distributed; (4) NAs dynamically form and reconfigure; (5) NAs activate simultaneously, and a reduced number of NA cells can trigger the activity of the entire NA; and (6) active NAs can activate other NAs and evolve in phase-sequences, possibly decoupled from sensory or internal events, providing the basis for complex cognitive processing (Sakurai, 1998). However, the temporal dynamics of the formation and dissolution of assemblies, which prevent the brain from converging on undesirable states such as a global synchronization, was not fully theorized by Hebb (Buzsáki, 2006). The original proposal has thus been extended to contemplate not only neurons with synaptic connections, but all cells presenting above chance interactions. In this way, the term NA has no absolute definition with respect to neuronal activity and can be associated with varying degrees of temporal precision, scale and internal structure (Russo and Durstewitz, 2017). Here, we will consider that NAs are formed by neurons that jointly increase their average firing rates for some period (Lopes-dos Santos et al., 2011, 2013). In any case, there is growing agreement that NAs will be better understood by inspecting their causal link with motor commands and other behavioral features (Buzsáki, 2010, and references therein). In particular, characterizing NAs within behavioral tasks is relevant for assessing the distributed role of cortical regions in distinct cognitive processes (Gutierrez et al., 2005; Hu et al., 2005; Carrillo-Reid et al., 2016; Dejean et al., 2016), for the understanding of the neural code (Winters and Reid, 2010; Russo and Durstewitz, 2017), and for the design of new brain-machine interfaces (Wessberg et al., 2000; Hochberg et al., 2006; Ifft et al., 2013; Shokur et al., 2013; Ramakrishnan et al., 2015).

In the context of studying distributed neural interactions and NA dynamics, active tactile discrimination tasks with rats are elucidative: it is a consolidated animal model (Bush et al., 2016), involving tactile stimuli and higher-order cognitive processes such as sensory-motor integration, attention and decision making, hence engaging several cortical areas in the processing of information (Stoeckel et al., 2003; Cao et al., 2008). These task aspects are not considered in passive or anesthetized scenarios (Szwed et al., 2003; Krupa et al., 2004). During active tactile discrimination, the primary somatosensory cortex (S1) activity is directly linked to vibrissae stimulation (Simons, 1978), and recent studies focus on establishing the relationship between S1 modulations and other cortical areas. For example, Pais-Vieira et al. (2013) showed that the anticipatory modulation of S1 is dependent on the primary motor cortex (M1). Zagha et al. (2015), in a sensory detection task, found competing ensembles of neurons in M1, whose opposing spiking patterns were related to mapping of sensory stimulus to motor commands.

In particular, there is a growing interest on understanding how multisensory information processing might relate to animal performance, i.e., how information integrated from multiple sensing modalities encodes behavior information (Driver and Spence, 2000; Sheppard et al., 2013; Semprini et al., 2016; Bieler et al., 2017a,b). There is growing evidence supporting interactions between S1 and the primary visual cortex (V1): for instance, Zangaladze et al. (1999) used transcranial magnetic stimulation (TMS) to disrupt the activity in V1 and verified an interference in the tactile discrimination used for spatial orientation during the free exploration of novel objects. Vasconcelos et al. (2011) recorded S1 and V1 simultaneously during rat blind exploration of distinct objects in a dark environment and reported a similar amount of task information in the neural response of both areas, which points to multimodal neural interactions. Moreover, Sieben et al. (2013) noticed that visual stimuli induced neuronal oscillations and modulated the power of S1 activity in a task where animals received simultaneous light flash and whisker deflection; and Sieben et al. (2015) reported a reduction on direct connections between S1 and V1 when animals received less stimulation in neonatal development, further supporting the hypothesis that information processing in primary cortical areas is distributed. Finally, Bieler et al. (2017a) reported higher phase-coupling when animals received bimodal (visual-tactile) stimulation.

In addition, experiments highlight the role of the prefrontal cortex (PFC) and the posterior parietal cortex (PPC) in tactile decision-making, considering that these areas are anatomically connected to a variety of sensory regions (Reep et al., 1994; Miller and Cohen, 2001; Behrmann et al., 2004; Murray et al., 2012; Licata et al., 2017). PPC engages in cognitive processing of attention (Behrmann et al., 2004; Stilla et al., 2007) and is involved in the spacial representation of the environment (Whitlock et al., 2008) as well as in the association between tactile and visual information in rats (Winters and Reid, 2010). The PFC, in turn, is associated to planning (Tanji and Hoshi, 2008; Martinet et al., 2011), working memory (Funahashi and Kubota, 1994; Riley and Constantinidis, 2015) and reasoning (Miller and Cohen, 2001) and in somatosensory information coding (Murray et al., 2012). In a model of PFC columnar organization, Martinet et al. (2011) showed that PFC single-cell activation is involved in spatial navigation planning, whilst Reid et al. (2013) reported impaired object recognition when PFC was lesioned.

In this work, we test the hypothesis that neurons distributed in long-range fronto-parieto-occipital networks participate in different NAs and are involved in active tactile discrimination. For that, we investigate the dynamic formation of NAs within and among the primary somatosensory (S1), primary visual (V1), prefrontal (PFC), and posterior parietal cortices (PPC), simultaneously recorded from seven Long-Evans rats during an active tactile discrimination task. Simultaneous recordings in the four aforementioned areas are, to the best of our knowledge, original in the literature, and can contribute to shed light on neuronal population interactions *within the same animal* engaged in a cognitive task. First, we show that neuronal activity in all four regions is modulated by tactile discrimination and reward collection. Then, using a neuronal decoder, we show that the majority of single neurons carry little information about the stimulus and reward, but when the whole population is considered, average decoding performance resembles or surpasses that obtained by animals in the task. Finally, we identify the dynamic formation of NAs during tactile discrimination, and show that assembly neurons contain more information about animal behavior than neurons that do not participate in the assembly. These findings further support that distributed neural interactions encode active tactile discrimination.

## 2. Experimental procedures

### 2.1. Subjects

This study was carried out in accordance with the recommendations of the National Institute of Health Guide for the Care and Use of Laboratory Animals, National Institutes of Health. The protocol was approved by the AASDAP Ethics Committee (CEUA 01/2013). We used seven adult male Long-Evans rats from IIN-ELS laboratory (Macaiba, Brazil), weighing 300–350 g at the start of training.

### 2.2. Multielectrode implants

The arrays were constructed with 50 μm tungsten wires, coated with Teflon (California Fine Wire Company) soldered in a printed circuit board and connected to a miniature connector (Omnetics Connector Corporation, Minneapolis, MN). The microelectrode arrays were designed for four different cortical regions: PFC, PPC, S1 and V1. The configuration of the arrays, detailed in Table 1, was established from stereotactic coordinates based on the atlas from Paxinos and Watson (2007). Two days before surgery, the rats were given access to water and food *ad libitum*. Following Wiest et al. (2007), the arrays were placed under deep ketamine (4,100 mg/kg i.p.) and xylazine (5 mg/kg i.p.) anesthesia in a stereotaxic head holder. The scalp was incised, the skin, periostium and dura mater over the interest region were retracted. After the craniotomy, each microelectrode array was lowered into specific regions (PFC, PPC, S1, and V1) and fixed with dental acrylic. The rats were then given 7 days of postsurgical recovery with free access to food and water.

**Table 1:** Steriotactic coordinates and arrangement of arrays used in the tactile discrimination task.

	**PFC**	**PPC**	**S1**	**V1**
AP (mm)	+2.20 to −0.40	−3.24 to −4.08	−2.04 to −3.24	−5.20 to −6.40
ML (mm)	1.40	1.8 to 3.3	5.2 to 6.4	3.8 to 5.0
DV (mm)	1.96	1.0	0.8 (layer 4)	1.67
			1.2 (layer 5)	
θ (degrees)	24	90	39	20
Configuration	1*X*8	1*X*4 + 2*X*6	4*X*4	4*X*4
Spacing (μ m)	200	300	400	400
Laterality	bilateral	unilateral	unilateral	unilateral

*In each neural implant, 64 microelectrodes were used, distributed in the 4 areas*.

### 2.3. Electrophysiological recordings

Signal acquisition was performed using the 64-channel OmniPlex Neural Data Acquisition System (Plexon Neurotechnology Research Systems, Dallas, TX), as described previously by Nicolelis et al. (2003). Spike signals were amplified (20.000–32.000x), filtered (400 Hz–5 kHz) and digitized at 40 kHz. Spikes from each electrode were classified on-line (Sort Client, Plexon) and validated off-line using spike-sorting software (Offline Sorter, Plexon Inc., Dallas, TX) according to the following cumulative criteria: (i) signal-to-noise ratio bigger than 2.5 verified on the oscilloscope, (ii) <0.1% of interspike intervals smaller than 1.0 ms and (iii) stereotype of waveform shapes, as determined by a waveform template algorithm and principal component analysis. Figure S1 details the spike sorting process. After the experiment, a dataset was created containing the neuronal spike activity of each individual cell identified. The precise times in which the animal placed its muzzle in the center nose poke defined the tactile stimulation events.

### 2.4. Histology

At the end of the last recording session, all rats were transcardially perfused with heparin (1 U/ml) in saline (0.9%), followed by 0.1 M phosphate buffered paraformaldehyde (4%, pH 7.4) under deep anesthesia with ketamine (80 mg/kg i.p.) and xylazine (10 mg/kg i.p.). Brains were removed immediately and fixed in 4% paraformaldehyde in PBS for 1 day, and then incubated in 30% sucrose in PBS for at least 2 days. After brain slice preparation, probe electrode locations were histologically verified by cytochrome c oxidase staining (Figure S2).

### 2.5. Behavioral task

**Figure 1:**
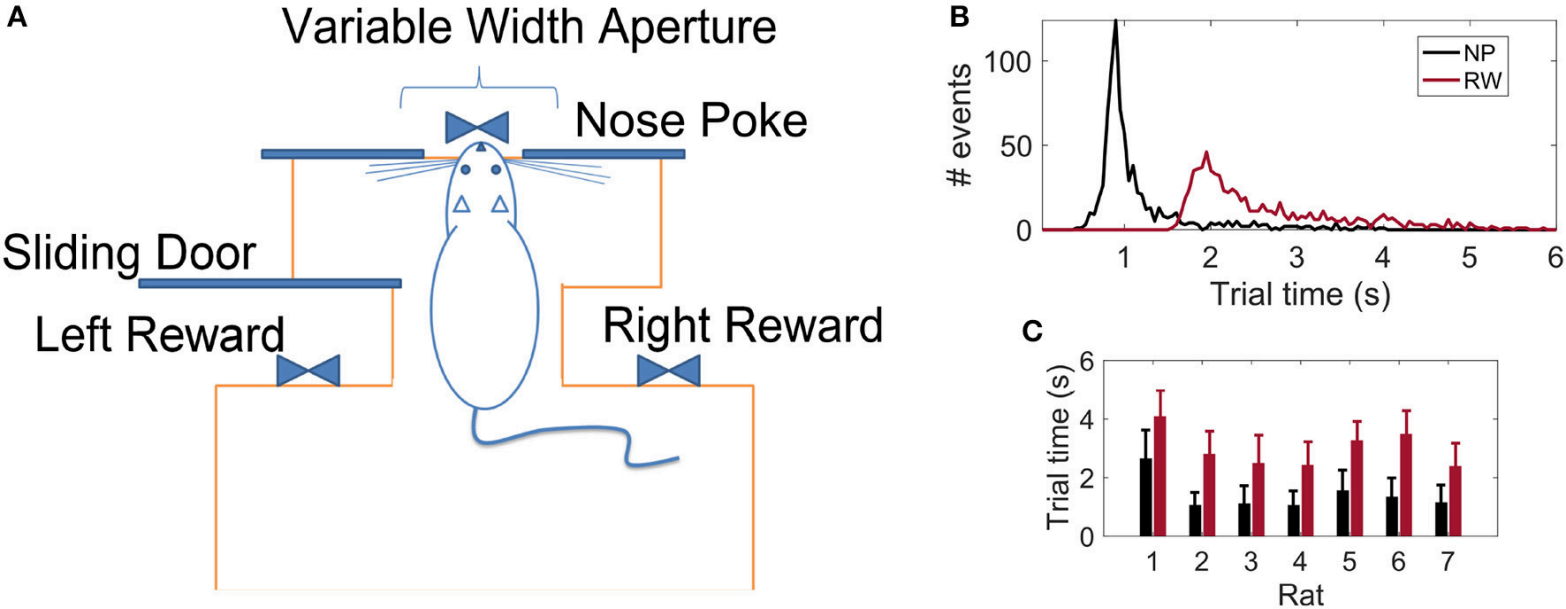
**(A)** Schematics of the behavioral apparatus: the animal has to discriminate between a wide or narrow aperture employing only their mystacial vibrissae in order to get a water reward (adapted from Krupa et al., 2001). **(B)** Distribution of reaction times considering 764 trials from all 7 rats: time to reach the Nose Poke (NP) or Reward (RW) sites with respect to the Central Door event (*t* = 0 s). **(C)** Reaction times per animal (mean ± std).

Following the work of Krupa et al. (2001), we trained seven *Long-Evans* rats in an active tactile discrimination task. The scenario consisted of a behavioral box (Figure 1A) with two chambers separated by a central door: the first chamber contained two opposite reward sites, the second, a corridor with wide (85 mm) or narrow (52 mm) apertures implemented by moving bars of variable length. The box contained infrared light beams that, when broken, marked the passage of the animal in the different parts of the scenario. At the beginning of each trial, the animal is confined in the first chamber. Upon opening of the central door, the rat enters the second chamber and has to move forward until its muzzle is placed in an orifice located at the opposite side of the chamber (center nose poke-NP). Whiskers are stimulated by the contact with the moving bars during the whole process of approaching and departing from the NP aperture, thus the NP time is defined as reference for the tactile stimulus event (Pantoja et al., 2007). As soon as the light beam in the NP orifice is broken, a water reward in the first chamber may be provided. If the aperture was wide, the reward is released on the right hand side of the chamber; if narrow, on the left. The water reward is provided only if the rat places its muzzle in the correct reward site. In this way, there is no accumulation of water reward due to wrong trials. Experiments were always carried in the absence of light to ensure that animals could use only their whiskers (and not their vision) to accomplish the task. Each animal was implanted with microelectrodes upon reaching at least 75% rate of success in 1 h of the task (≈ 150 trials in one recording session).

This task was originally proposed by Krupa et al. (2001) and animal behavior was further studied in subsequent works (Krupa et al., 2004; Pantoja et al., 2007; Wiest et al., 2010; Vasconcelos et al., 2011; Pais-Vieira et al., 2015). The behavioral apparatus design results in stereotypical animal trajectories over the course of the trial, with whiskers sampling the aperture for a few hundred milliseconds. Experiments comprising whisker removal, lesions of the barrel cortex, and sectioning of the facial nerve confirmed that intact whiskers and S1 cortex are necessary for accurate discrimination, though whisker movements are not. No other sensory cues other than tactile could be used to accurately solve the task.

### 2.6. Data analysis

All of the analyses detailed below relate to the behavioral task described above. For each animal, we built a dataset of spike activity from PFC, PPC, S1, and V1. This dataset also includes the precise time in which the central door opened, when animals placed their muzzle in the nosepoke orifice, and when they collected the water reward. To assess neuronal activity over time, we used raster plots, peri-stimulus time histograms and mean firing-rate analyses. To highlight the distributed neuronal interactions, neuronal assemblies were characterized prior to and when the animals were engaged in the active tactile discrimination task. Finally, information content in spike activity was estimated using a support vector machine decoder.

#### 2.6.1. Peri-stimulus time histograms and mean firing rate

Neuronal data was binned using a sliding 10 ms time-window (no overlap). The baseline neural activity is defined as the [−3 −2] s period prior to the NP event. Mean neuronal firing rate over time (MFR) was calculated from spike trains in 50 ms time windows (no overlap).

#### 2.6.2. NAs characterization

**Figure 2:**
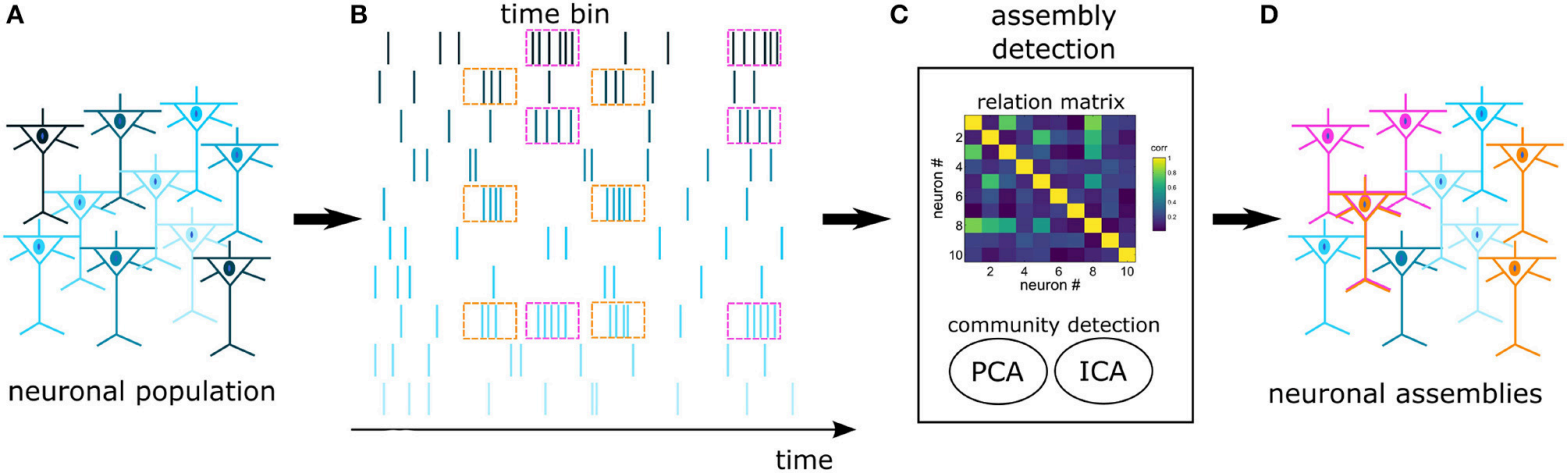
Schematics of NA detection: Spikes are recorded from a pool of cortical cells **(A)**, forming a set of spike trains **(B)**. The trains are binned and fed into an assembly detection algorithm **(C)**. A relation matrix is built based on the covariance of spike trains, followed by a PCA+ICA community detection algorithm. The assembly detection algorithm identifies neurons with joint activations (pink and orange) and independent neurons (blue) **(D)**. In this work, functional NAs are defined by time-specific coherent modulations in the firing rate. Notice that one neuron may participate in more than one NA.

In an experimental pool of recorded cells there is a high likelihood that many cells won't engage in NAs. As a result, we opted for an algorithm that is efficient in discriminating cells with independent activity. Several works have addressed the problem of extracting NAs from spike trains (Kreuz et al., 2007; Humphries, 2011; Lopes-dos Santos et al., 2013; Billeh et al., 2014), however, within this set of algorithms, only the proposal of Lopes-dos Santos et al. (2013) is not hard-clustering by design, i.e., it does not require that each neuron belongs to a NA. This algorithm considers that functional NAs may encompass cells from multiple regions, interacting through time-specific coherent modulations in their firing rate. We emphasize that other algorithms may consider distinct NA definitions, as reviewed by Russo and Durstewitz (2017). The next paragraphs describe with more details this specific approach (Lopes-dos Santos et al., 2011, 2013), and a schematics is provided in Figure 2.

Initially, the interactions between spike trains of pairs of neurons are quantified and stored in a relation matrix, in this case the covariance matrix computed from the mean firing rate of the neurons. Then, we identify communities of neurons (NAs) within this covariance matrix with PCA. The reasoning is as follows: First, the principal components (PCs) **p_i_** are calculated from the eigenvectors *P* = [**p_1_**, …, **p_n_Neurons__**] of the relation matrix. The next step is to evaluate whether interactions between spike trains are strong enough to be distinguishable from spurious temporal associations. Considering spike trains from a population of neurons and the respective covariance matrix, the question is to whether a given coefficient in that matrix denotes a statistically significant correlation between a pair of neurons, i.e., one would have to test for the null hypothesis that spike trains are independent random variables. To this end, we define a statistical threshold. One way of obtaining such threshold value is to use surrogate methods, but, as noted by Lopes-dos Santos et al. (2011), this may lead to a computational burden. However, Peyrache et al. (2010) have shown that the analytical threshold following from the Marčenko-Pastur distribution can be used as statistical threshold. Marčenko and Pastur (1967) have shown that the autocorrelation matrix calculated from *N*_*lines*_ independent vectors, with length *N*_*cols*_ has eigenvalues (λ) following the analytic distribution *p*(λ), described in Equation 1, where $q=\frac{N_{cols}}{N_{lines}}$ and σ^2^ refers to the variance of the elements.

(1) $$\{\begin{array}{l} p(\lambda )=\frac{q}{2\pi \sigma^{2}}\frac{\sqrt{(\lambda_{max}-\lambda )(\lambda -\lambda_{min}})}{\lambda }\text{ where}\\ \lambda_{min}<\lambda <\lambda_{max};\text{ and}\\ \lambda_{min}^{max}=\sigma^{2}{\left(1\pm \sqrt{\frac{1}{q}}\right)}^{2} \end{array}$$

Given that the probability of having independent vectors with eigenvalues > λ_*max*_ is zero (Lopes-dos Santos et al., 2013), Equation 1 can be used as a threshold to NA formation. If, for example, a group of neurons within a population constitutes a NA (i.e. the group neurons jointly increase their average firing rates for some period), its firing rate profile as captured by the covariance matrix will result in a PC whose associated eigenvalue is greater than the limit defined by Equation 1; similarly, if neurons within a population are independent, all eigenvalues λ from the covariance matrix lay within the distribution (λ_*min*_ ≤ λ ≤ λ_*max*_). Therefore, the number of detected assemblies *nda* is numerically equal to the number of eigenvalues λ of the relation matrix *S* that are greater than λ_*max*_, and the principal components associated to eigenvalues that crossed the threshold established by the Marčenko-Pastur distribution can be considered as statistically significant in the process of identifying NAs.

If in a neural population the NAs do not share neurons, then, for each eigenvalue that exceeded λ_*max*_, the weights of the corresponding PC, given by the elements *w*_*k*_, *k* = 1, …*n*_*Neurons*_ of a PC **p_i_**, indicate the neurons that belong to the NA: ideally, NA members have similar, nonzero weights while independent neurons have null or near zero weights (Lopes-dos Santos et al., 2013).

However, there are limitations when NAs do share neurons: the PC weights distribution is scattered and the identification of NA members is no longer possible (Lopes-dos Santos et al., 2011, 2013). To handle that, Independent Component Analysis (ICA) is added as an additional step. Following Lopes-dos Santos et al. (2013), we used the fastICA algorithm (Bingham and Hyvärinen, 2000). ICA separates statistically independent components of a signal, assuming that the signal results from the linear combination of several independent sources. The rationale is as follows: the collection of spike trains used to calculate the relation matrix can be projected on each of the PCs whose eigenvalues are > λ_*max*_ (PCs that relate to NAs); iteratively, ICA acts on the PC weights *w*_*k*_ to produce maximally independent projections; finally, to identify which neurons belong to a given NA, a threshold is applied to the modified weight pattern $w_{k}^{ICA}$ of each NA-related PC–PC weights larger than this limit point at assembly neurons. Here, the threshold is defined as the mean value of $w_{k}^{ICA}$ added to one standard deviation.

#### 2.6.3. Support vector machine classifier

To estimate the amount of information about reward side that is contained in neural activity over time windows, we implemented a support vector machine (SVM) classifier. The SVM classifier maps a feature vector (in our case, mean firing rate of neurons) into labels (left or right reward side). For each trial, starting at *t* = −4 s and at every 350 ms time window (50 ms time step), the feature vector was determined by concatenating the mean firing rate of neurons from the given cortical region in seven consecutive 50 *ms* windows. Parameters were chosen based on Pantoja et al. (2007), Wiest et al. (2010), Vasconcelos et al. (2011), and Pais-Vieira et al. (2015) and analyses were robust to minor variations on this choice. The label of each feature vector was either “0” (left side reward) or “1” (right side reward). Following a three-fold cross validation procedure, we trained the SVM classifier using the lib-svm toolbox (Chang and Lin, 2013). A radial basis function (RBF) kernel was used and the best C/gamma parameter combination was iteratively searched in a base-2 logarithmic grid from –18 to 18. Simply put, parameter C influences error threshold and classifier stability whilst gamma relates to the decision boundary surface.

### 2.7. Statistical analysis

**Figure 3:**
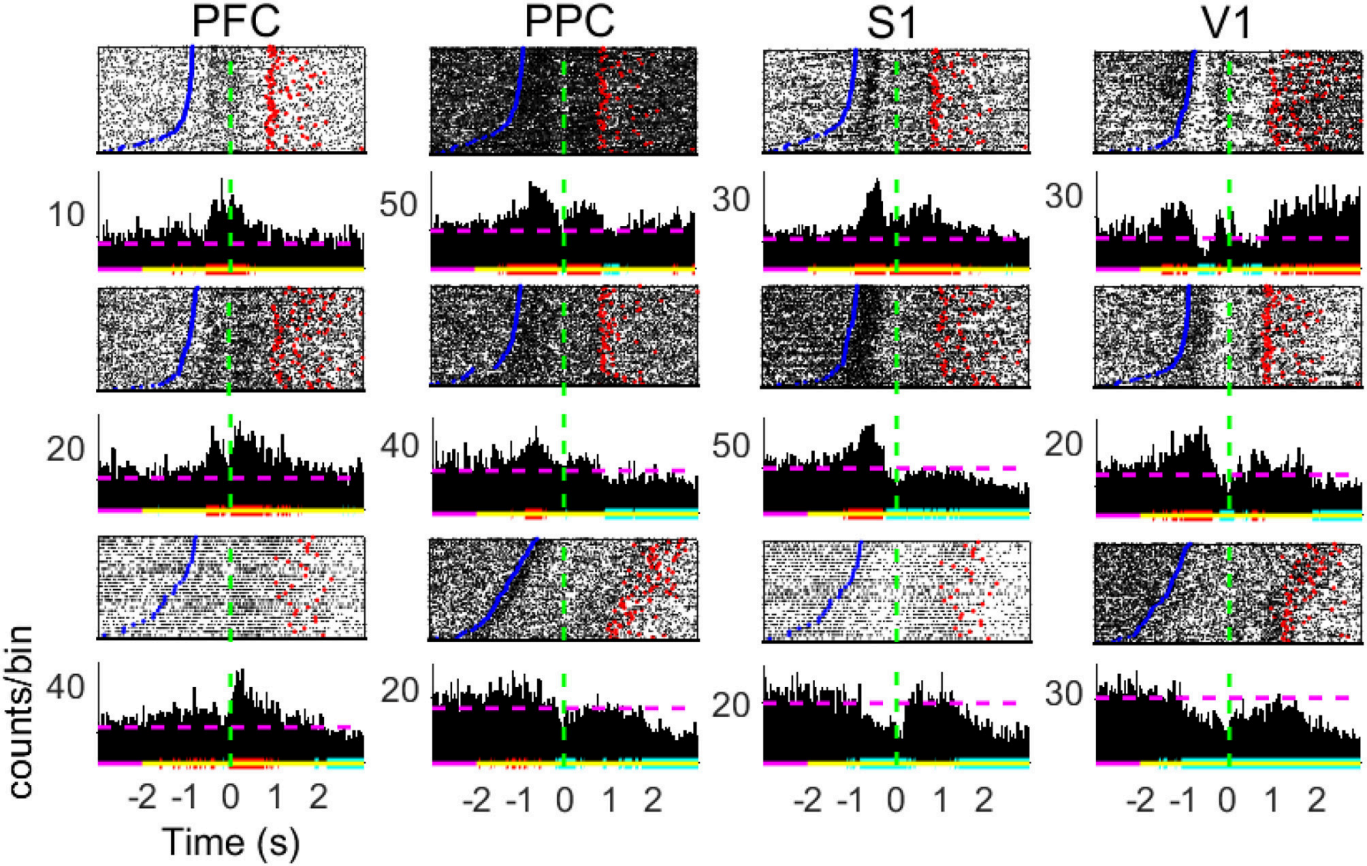
Peri-stimulus time histograms of neuronal responses of single cells. Panels show 3 representative neurons from diverse subjects in each of the brain regions recorded. Neuronal data was binned using a sliding 10 *ms* time-window (no overlap). The baseline neural activity is defined as the [−3 −2] s period. Red (blue) horizontal lines indicate significant increase (decrease) in firing modulations; purple (yellow) lines relate to the baseline (analyzed) period; dashed horizontal lines depict the MFR in the whole period. Time *t* = 0 mark the moment rats reach the NP (dashed green horizontal line). Blue points indicate the beginning of trials (Central Door) and red points indicate the water reward delivery.

Unless stated otherwise, results are reported as means ± standard error of the mean (SEM) across the total number of neurons. In the peri-stimulus time histograms (Figure 3), a method based on cumulative summed spike counts (Gutierrez et al., 2005; Wiest et al., 2005) was used to assess significant deviations from baseline neural activity, defined as the [−3 −2] s period prior to the NP event. To assess the difference in decoding accuracy between NA neurons and randomly chosen neurons, we used a non-parametric test based on bootstrapping (function *statcond* from the EEGLAB toolbox Delorme and Makeig, 2004). All data were analyzed using MATLAB (The Mathworks, Natick, MA).

## 3. Results

The number of recorded units in each cortical area and the number of successful trials from each animal's dataset are presented in Table 2. Table 3 include task performance. Animal reaction times and movement times over the trial period, is presented in Figure 1B considering all rats, and in Figure 1C for each rat.

**Table 2:** Number of recorded units in each brain region and total number of successful trials.

	**Rat 1**	**Rat 2**	**Rat 3**	**Rat 4**	**Rat 5**	**Rat 6**	**Rat 7**
PFC	23	17	27	16	23	20	28
PPC	16	17	22	20	10	9	23
S1	14	8	12	9	19	15	12
V1	26	22	20	21	43	38	23
S. Trials	102	118	204	254	190	196	198

**Table 3:** Performance summary (%).

	**Task**	**NA**	**Peak population**				**Peak single neurons**				**Avg single neurons**			
			**PFC**	**PPC**	**S1**	**V1**	**PFC**	**PPC**	**S1**	**V1**	**PFC**	**PPC**	**S1**	**V1**
Rat 1	82	85	85	92	92	96	85	77	88	88	58	56	60	58
Rat 2	82	93	80	80	100	97	87	77	87	87	58	49	63	62
Rat 3	80	100	100	73	87	96	100	73	87	92	64	54	57	61
Rat 4	74	100	100	98	81	98	98	94	78	88	65	61	54	67
Rat 5	95	92	88	69	85	79	83	71	77	83	55	54	55	57
Rat 6	92	92	90	70	78	94	86	76	80	86	53	57	55	56
Rat 7	79	100	100	84	96	92	98	80	98	88	63	55	60	64

*Animal behavioral performance in the task (second column) and peak SVM decoding performance for each region considering the following inputs: (i) NA neurons (third column); (ii) all population neurons (Figure 5); (iii) individual single neurons (Figure S5). Peak single neurons group shows the best decoding performance obtained from a given single unit whilst the average single neurons group displays the average of all single neurons decoding performance*.

### Neuronal activity is modulated by tactile discrimination

To investigate the occurrence of distributed neural interactions during the active tactile discrimination task, we begin by studying the neural population firing rate dynamics over time. Firing rate variations, though a simple coding strategy, is a hallmark of neuronal interactions (Jacobs et al., 2009; Marsat and Maler, 2010; Rolls and Deco, 2010; Zuo et al., 2015) and underlie NA formation (see Section 2.6.2).

The peristimulus time histograms (PSTHs, Figure 3) reveal a great diversity in individual cell activity profile within the four cortical regions of interest (neurons either present an increase in MFR, a decrease, a combination of both, or are unresponsive to the task–see Table S1). From Figure 3, we note statistically significant firing rate modulations with respect to the baseline interval in different periods of the task, including the reward period. These diverse neuronal responses next to the tactile stimulus event is suggestive of NA formation and may be related to information processing.

**Figure 4:**
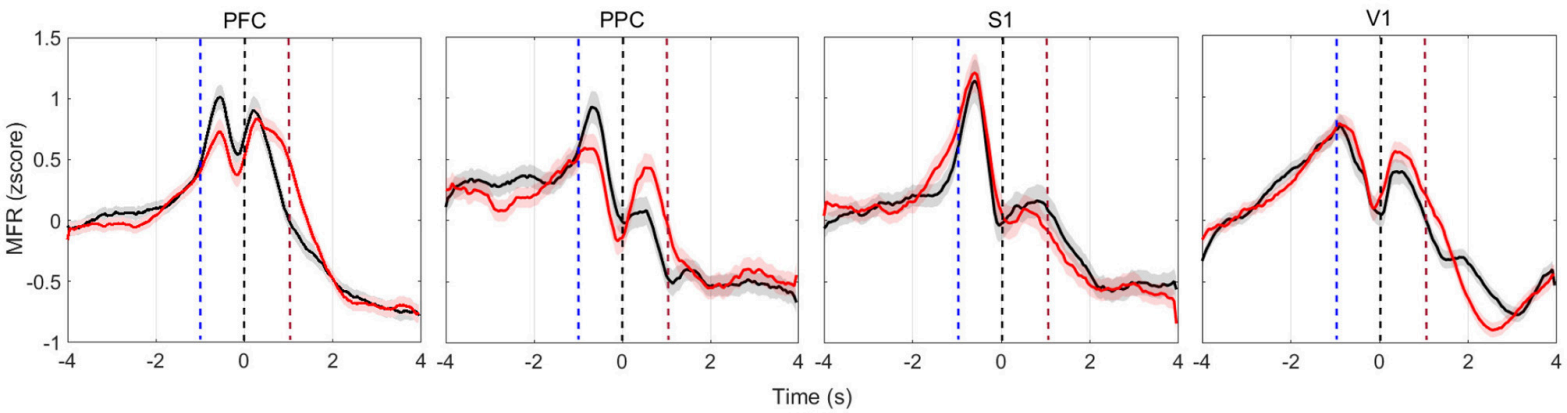
Multiphasic neurons mean firing rate (bin = 50 ms, no overlap, z-scored) of all animals in each of the four recorded cortical areas. Red (black) lines depict the activity when reward was at the left (right) hand side. Time *t* = 0 s marks the moment rats reach the NP. Shaded regions relate to the standard error of the mean (calculated across the total number of recorded cells, described in Table 2). Curves are smoothed with a 10-point moving average filter. Dashed blue (red) vertical lines indicate the approximate beginning of trials (reward) time.

PSTHs depict single-neuron responses. To obtain a population perspective, we assessed the mean firing rate dynamics within the four cortical regions recorded. To reduce discrepancies, we only averaged neurons of similar profile (increased, decreased, or both). Figure 4 shows the MFR of neurons presenting both increased and decreased responses (named multiphasic units), the most likely type of modulation in the cortical layer from which we recorded (Pais-Vieira et al., 2015). All cortical areas modulate their MFR: neuronal activity peaked in the four brain regions upon vibrissae stimulation, soon before rats reach the NP, similarly to previous findings (Pais-Vieira et al., 2013). The increase and decrease pattern in MFR over time shared similarities for the wide and narrow apertures, but there is a clear difference in the [−2 2] s window around NP (Figure S3).

The PSTH and MFR results in all recorded regions suggest that modulations in firing rates comprise event-related information. To test this hypothesis, we proceeded by implementing a SVM classifier to estimate the amount of information about reward side (and hence tactile discrimination information) that is contained in firing rate modulations.

### Event-related information is distributed in the brain

**Figure 5:**
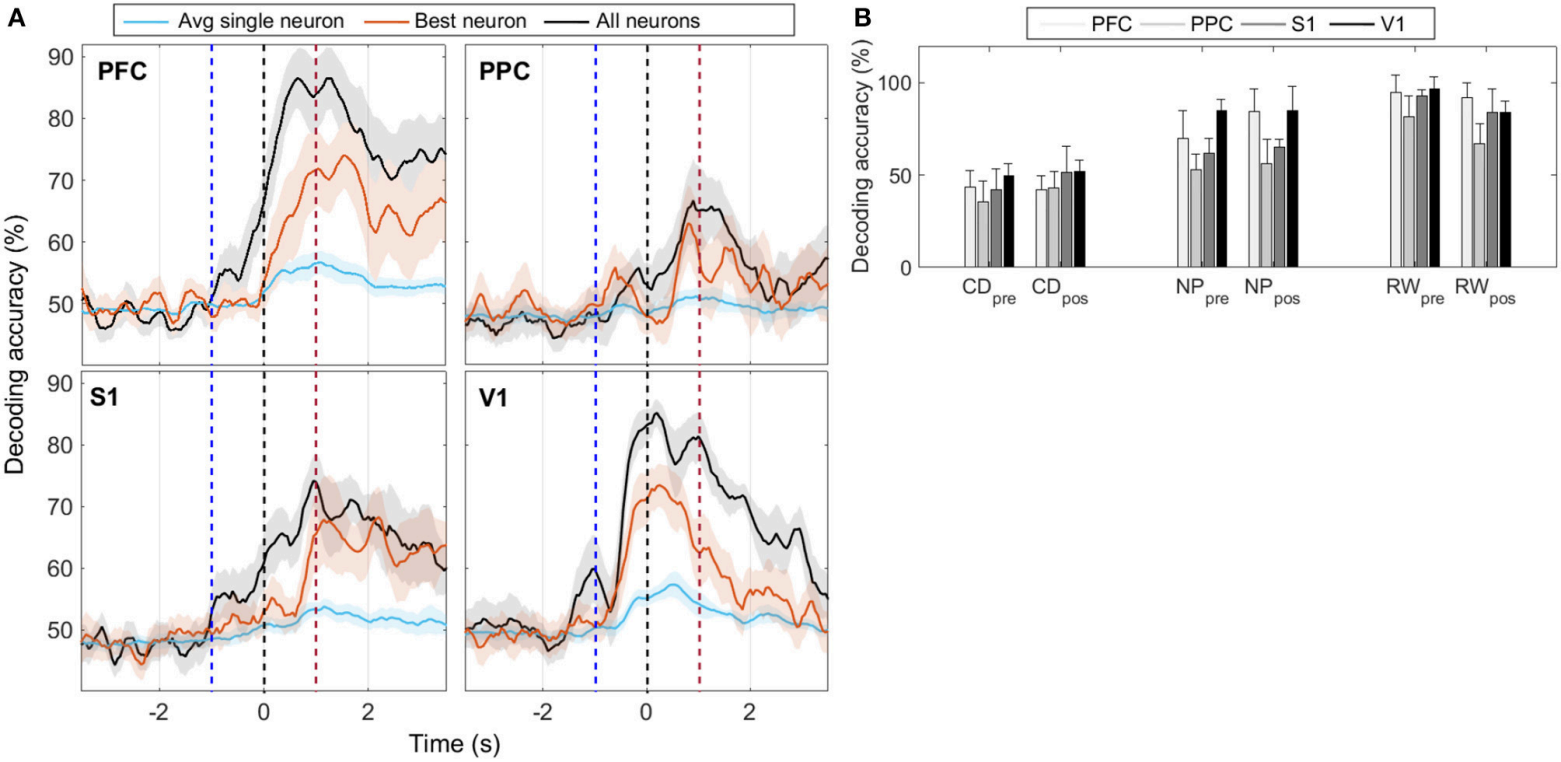
**(A)** Mean animal response predicted with a SVM decoder. The decoder is fed either with the activity from all recorded cells within each cortical region (black), or with the activity from each neuron, individually. Average (best) single-neuron decoding accuracy is shown in blue (red). For each trial, the decoder mapped seven consecutive 50 ms windows of spike activity (50 ms time step) into left or right reward side. Labeled time points indicate the ending of the sliding time windows. Curves are smoothed using a 5-point moving average filter. Time *t* = 0 s marks the moment rats reach the NP. Shaded regions relate to the standard error of the mean (calculated across the total number of animals). Dashed blue (red) vertical lines indicate the approximate beginning of trials (reward) time. **(B)** Mean ± std of animal response predicted with a SVM decoder using the neural activity from a 350 ms window immediately prior to or after the central door (CD), nose-poke (NP), and reward (RW) events.

For each cortical region recorded, we built SVM classifiers (Section 2.6.3) to predict reward side over time both from single-neuron and population responses. The results, shown in Figure 5 and Figures S4–S6, indicate that population activity contains more tactile information than single neurons alone. We note, however, that decoding performance was not uniform across cortical regions and some animals had single-neurons whose responses had information content similar to that of the neural population (Table 3). In accordance with MFR differences (Figure S3), decoding performance peaked soon after the nose poke (*t* = 0 s) and, particular to PFC, it was sustained until reward collection. PFC and V1 presented the highest decoding accuracy levels, however this may be attributed to the unequal number of neurons recorded in each region (see Figure S6). Nevertheless, classifiers had greater than chance performance in all regions. This suggests that event-related information is distributed within PFC, PPC, S1, and V1.

We have shown that task-related variations in neuronal MFR are widespread in all four regions recorded and the SVM classifier analysis reveal that tactile discrimination information is present in neural responses. Next, we assess whether these informative MFR modulations relate to groups of co-activated neurons–neuronal assemblies.

### NAs have been consistently characterized during tactile discrimination task

We characterized NAs within and among PFC, PPC, S1, and V1. To calculate the relation matrix (Section 2.6.2), spike trains from each neuron were obtained by concatenating 3 s data segments centered on NP time and MFR was calculated using 10 ms time bins. Two important time frames are assessed in this analysis: 1) when the central door is closed and the trial has not yet begun, and 2) when the animal is engaged in the active tactile discrimination task ([−1.5 1.5] seconds around NP event). These time windows were chosen because they capture two clearly different behavioral states and hence could robustly reveal the emergence and vanishing of neural assemblies regardless of minor differences in animal movement times.

**Figure 6:**
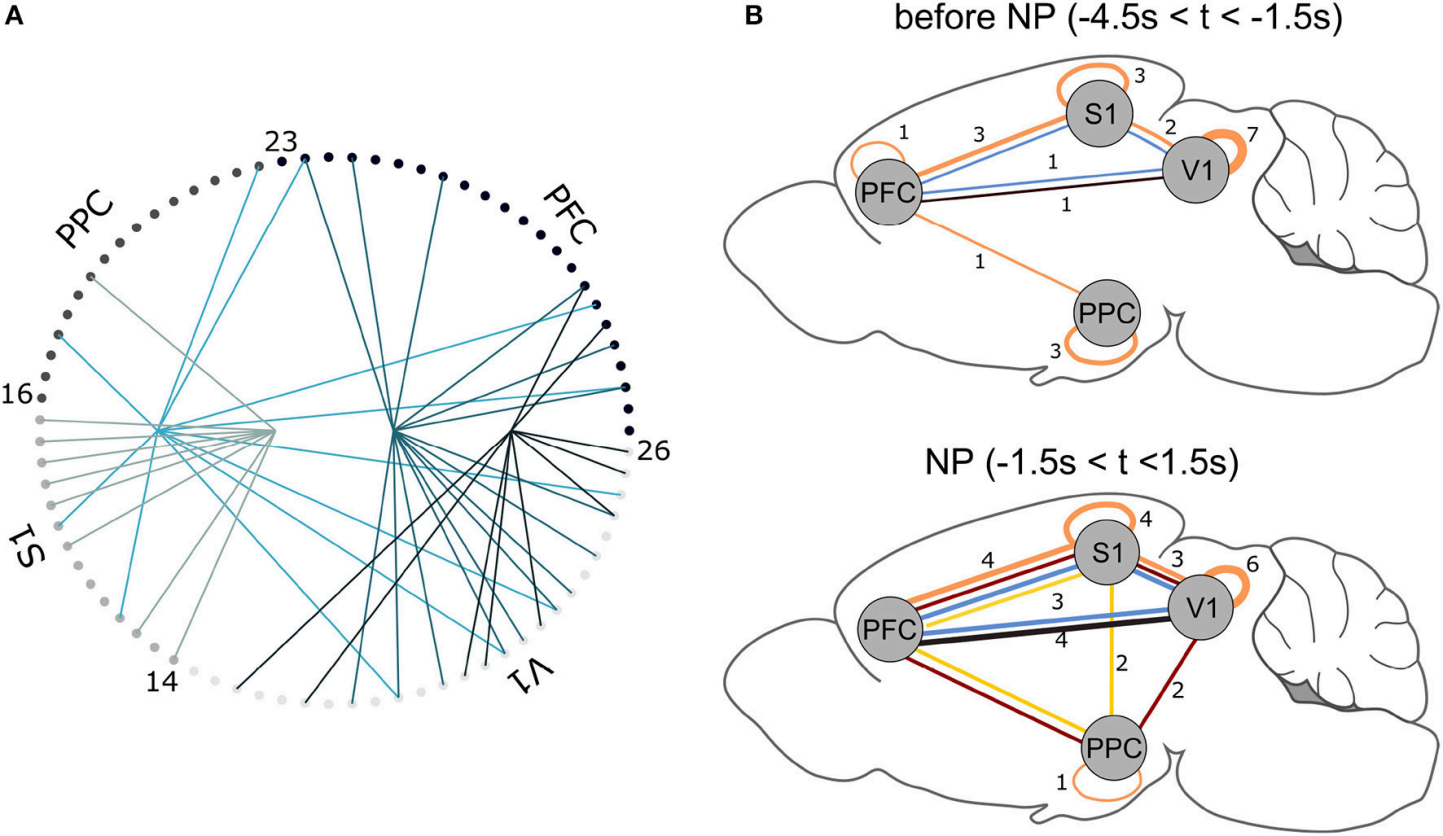
**(A)** NAs found for Rat 1. For each trial, 3 s data segments were selected centered on NP. Each small circle corresponds to one recorded neuron (see Table 2); lines of equal color connect neurons that belong to the same NA. **(B)** NA summary for all animals. Lines connect cortical areas where at least 10% of cells were engaged in the same (color-coded) NA. The thicker the connections, the greater the number of animals presenting NAs between the indicated cortical regions. For example, in the NP time window, 3 animals presented functional interactions within PFC, S1 and V1 (blue NA); before NP, only one animal displayed this interaction. Brain template adapted from Töle (2014).

Figure 6A shows the results for one animal. Notice that NAs are of different cardinality and comprise neurons from multiple cortical regions. Also, there are neurons that belong to more than one assembly. This is in line with results found on the PSTHs (Figure 3) and on the MFR analysis (Figure 4), which show significant neuronal firing modulations with respect to the NP event in all of the four regions studied. Moreover, the decoding accuracy results (Figure 5 and Figure S5) suggest single-neurons are hardly sufficient to discriminate between wide and narrow apertures, which further supports the hypothesis of interactions between primary sensory and associative areas within the tactile discrimination task.

In all animals, the NA characterization pipeline consistently pointed to NA formation before and after the time window the animal engaged in the active tactile discrimination task (Figure 6B). Functional connections between cortical areas, represented by NA formation, vary within animals over time–tactile discrimination change the populational functional connectivity profile. Before tactile discrimination, 3 out of 7 animals presented functional interactions solely within the same cortical regions; only one animal had a NA comprising 3 regions. Upon tactile discrimination, functional connections emerge and the network changes its configuration: 6 out of 7 animals had NAs comprising neurons from 3 or 4 regions; all animals had neurons from at least 2 regions engaged in NAs. Thus, tactile discrimination onset promotes interactions between primary sensory (S1 and V1) and associative (PFC and PPC) areas.

When characterizing NAs with methods based on PCA and ICA, the length of the time-series and choice of time bins interfere on the identification of co-activation patterns of neural activity (Lopes-dos Santos et al., 2013), thus the results should be interpreted with care. To provide further evidence of behavior-related distributed neuronal interactions, we repeated the NA characterization but this time we considered a shorter, 1 s time window (a) prior to trial beginning (central door opening), (b) centered on the NP event, and (c) centered on reward delivery. Results are reported in Figure S7. Likewise the previous result, engagement in tactile discrimination promotes functional connections and a larger number of NAs is detected.

Finally, we conclude the analyses by assessing whether the identified NAs improve our capability of predicting animal behavior in comparison with a randomly chosen group of neurons.

### NA neurons encode more information about animal behavior than random neurons

**Figure 7:**
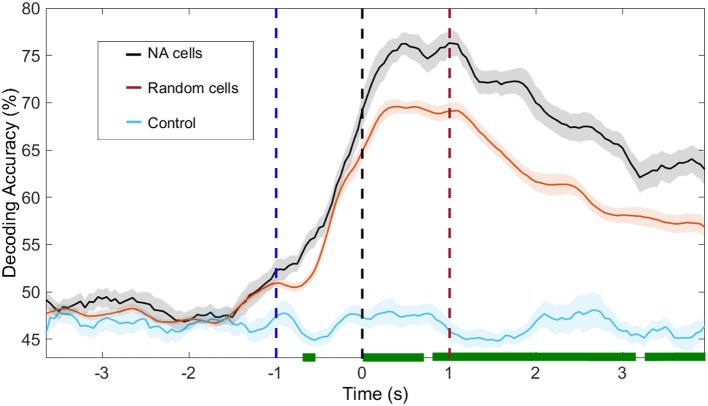
Decoding accuracy of NAs and random neurons. In black, we show the decoding accuracy using solely neurons from the NAs identified with ICA; in blue, the same cells were used but labels of animal response (left/right) are shuffled. In red, we use a random group of neurons out of the total population, containing the same number of cells as the identified NAs. We recall that no further restriction is imposed, i.e., the selection of cells belonging to other NAs, which also significantly modulate their firing rate, is possible. The shuffling process was repeated 100 times. In green, we depict the moments in which the decoding accuracy in NA and random cells are significantly different (*p* < 0.05). Time *t* = 0 s marks the moment rats reach the NP. Dashed blue (red) vertical lines indicate the approximate beginning of trials (reward) time.

To investigate the information content in assembly neurons, we selected two groups of cells: the first contained only neurons identified as belonging to NAs; the second, a random group of cells, containing the same number of neurons as the identified NAs, selected from the overall population. The analyses followed the same procedure described in Section 15. Results are presented in Figure 7.

One second before the nose poke (*t* < −1*s*), our ability to predict animal behavior from neuronal activity, regardless of cell group (NA neurons or random neurons), is at chance level. Immediately before the NP (−1 < *t* < 0 s), upon vibrissae stimulation, decoding accuracy increases and peaks at approximately *t* = 1 s. From this moment onward, decoding accuracy from NA cells is higher than that obtained from random cells (*p* < 0.05, bootstrap test).

## 4. Discussion

There is growing evidence that neural interactions are distributed (Nicolelis et al., 1997; Harris, 2005; Buzsáki, 2010). Thus, the study of neuronal assemblies (NAs) is relevant as it furthers the understanding of functional interactions and information processing in the brain. The characterization of NAs is still debatable (Lopes-dos Santos et al., 2013; Litwin-Kumar and Doiron, 2014; Russo and Durstewitz, 2017), though numerous studies have provided insights on the link between neural population dynamics and animal behavior (Terada et al., 2013; Siegle et al., 2014; Romano et al., 2015; Campagner et al., 2016; Dejean et al., 2016). In this work, we recorded 553 neurons from PFC, PPC, S1 and V1 of rats engaged in an active tactile discrimination task. Importantly, our analyses exploit simultaneous recordings from areas involved in primary sensory processing (S1 and V1) and higher-order cognition (PFC and PPC). Our results reveal task-related distributed neural activity and the formation and dissolution of NAs, which are suggestive of distributed neural interactions. We show that neural population responses, and in particular NA neurons, contain more tactile information than random groups of neurons. There are, however, single neurons scattered among the recorded regions with information content comparable to that of groups.

The first set of analysis, PSTH (Figure 3) and MFR (Figure 4), suggests that all of the selected cortical areas may be enrolled in the discrimination of tactile information: the overall neuronal activity presented significant firing modulations in the vicinity of the tactile discrimination period. Changes in neuronal activity are more prominently observed shortly before the nose poke (NP), when vibrissae stimulation and anticipatory effects are in place, and after the nose poke, which may be either due to the decision making process and reward collection, or to sensory-motor integration (Zangaladze et al., 1999; Krupa et al., 2004; Vasconcelos et al., 2011; Sieben et al., 2013; Namboodiri et al., 2015). The origins of anticipatory modulations were investigated by Pais-Vieira et al. (2013) in thalamo-cortical loops during the same tactile discrimination task. These authors indicate that anticipatory activity depends on top-down effects generated in part by motor gating. Therefore, despite the stereotypical animal behavior observed in this task, brain dynamics may be sensitive to task performance levels but also to the different behavioral strategies developed by animals (Nienborg et al., 2012; Sachidhanandam et al., 2013; Manita et al., 2015; Siegel et al., 2015; Yang et al., 2015). Further work should explore the relationship between neural dynamics and task performance.

The decoding accuracy of animal response from their neuronal firing modulations (Figure 5) increased above chance levels from the moment whiskers contacted the variable length bars to the collection of reward. In PFC and PPC, decoding accuracy peaked shortly after tactile stimulus, which is in agreement with the roles of these cortical areas in attention, planning and reasoning (Miller and Cohen, 2001; Behrmann et al., 2004; Stilla et al., 2007). For the primary cortical areas, the decoding accuracy for V1 peaked before S1, and reached higher values (mean of 85% vs. mean of 75%), but this difference may be attributed to the different number of neurons recorded in each area (Figure S6). In this task, sustained decoding accuracies after the tactile stimulation have been noted before in S1 and V1 (Krupa et al., 2004; Pantoja et al., 2007; Wiest et al., 2010; Vasconcelos et al., 2011), and here we provide the first report that the same occurs in PFC and PPC. Previously, Gardner et al. (2007) has reported anticipatory activity in PPC preceding the tactile responses during active touch in monkeys. From Figure S3, we observe markedly differences in spike rate between the wide and narrow scenarios from NP to reward, which explains the decoding profile. This sustained firing rate differences observed in all regions, but in particular in PFC, may be linked to the temporal evolution of the decision-making process required for tactile discrimination (Romo et al., 1999; Hernandez et al., 2002; Brody et al., 2003; Sreenivasan et al., 2014). Finally, the task studied comprises several aspects of cognitive processing, such as attention, decision making and sensory discrimination, thus further work is needed to clarify the neural information dynamics between primary sensory and associative areas in the context of this task.

Grand populational averages of MFR may mask the functional interactions in neural subgroups. The NAs characterization, performed by PCA associated with ICA, identified groups of neurons with joint activations beyond chance levels (Figure 6). From this characterization, we inferred a functional relationship map between the brain regions studied, before and during tactile stimulation (Figure 6B). Before NP, we noticed that the majority of functional network connections were restricted within single areas. At NP, functional connections emerged. The NAs comprising neurons from S1 and V1, found in 6 out of 7 animals, is in line with the findings of Vasconcelos et al. (2011), who reported distributed information processing in cortical primary sensory areas. PPC engaged in more NAs comparing the period prior NP and during NP, further supporting its role in multisensory integration (Winters and Reid, 2010; Harvey et al., 2012; Raposo et al., 2014). PFC established functional relationships with S1 in all of the recorded animals and with V1 in 6 out of 7 animals. Considering decision making tasks, this supports the observed central role of PFC in communicating with primary areas, engaged either in information coding (Murray et al., 2012) or planning and reasoning (Miller and Cohen, 2001). In addition, PFC is spatially distant from the other aforementioned cortical regions and their engagement in NAs during the discrimination task is an additional indicative that information processing is distributed in the brain (Nicolelis et al., 1995; Nicolelis, 2005; Winters and Reid, 2010; Guo et al., 2014). Finally, our results are aligned with the asynchronous convergence hypothesis (Nicolelis et al., 1995; Nicolelis, 2005), i.e., that active tactile discrimination results from the dynamic interplay of multiple descending, ascending, and local afferents that converge asynchronously on neurons located at each stage of the trigeminal pathway.

When assessing distributed interactions and the formation of NAs, the information content present in the neural population in comparison to that present in single-neurons provides further insights on the relationship between neural dynamics and behavior. As noted by others (Krupa et al., 2004; Pantoja et al., 2007; Wiest et al., 2010), the average single-neuron is minimally informative about aperture width whilst population decoding approximates or even exceeds animal performance (see Figure 5, Figures S4, S5, and Table 3). NA neurons contained more information about reward side than random groups of neurons, as demonstrated in Figure 6. However, there are highly informative single-neurons (Figure 5 and Figure S4). Bearing in mind subsampling distortions, we note that even if a single neuron presents a high decoding performance, Hebb emphasized that assemblies would evolve over time as phase sequences and these would facilitate cognitive acts, including motor behavior. Thus, according to Hebb, phase sequences would carry more information than neuronal firing rates (Almeida-Filho et al., 2014). Also, Buzsáki (2010) remarks in his comprehensive review of NA activity that determining the size of a NA is challenging and there is ample evidence that the contribution of single neurons to a given NA is skewed, i.e., a small number of assembly members may be as informative (from the decoder point of view) as a much greater number of active cells. However, one single neuron may not suffice to discharge downstream neurons - a key theorized role for NAs.

In addition to S1, we recorded from primary visual cortex because of previous reports on its involvement in cross-modal responses during tactile tasks (Zangaladze et al., 1999; Sadato et al., 2004; Vasconcelos et al., 2011). To prevent animals from using visual or auditory cues to perform the task, the behavioral apparatus was located inside a sound-attenuating and light-proof isolation box, previously described by Krupa et al. (2001). In the same task described here, the authors have shown that animal performance drops to chance when whiskers are cut. Besides, we only used infrared light during our recording sessions because rats do not have red nor infrared vision (Szél and Röhlich, 1992; Jacobs et al., 2001). Also, using the same behavioral apparatus, Vasconcelos et al. (2011) have shown that V1 neurons are recruited for tactile processing in the absence of visible light. In our work, modulations in firing rate are observed in V1 seconds before the rat samples the bars with whiskers (Figures 3, 4), but differences in firing rate due to narrow or wide apertures (Figure S3) only appear after tactile stimulation. James et al. (2006) showed that visual and haptic stimulus caused similar time course activation of the lateral occipital cortex and argue that there is mounting evidence that visual and haptic systems share the same neural substrate in encoding the structure of objects. Also, Amedi et al. (2002) report an overlap between visual and haptic processing in regions regarded as purely visual. Furthermore, Saito et al. (2006) showed that tactile stimuli activate V1 in well trained players of the chinese game of Mah-Jong. This is in line with Zangaladze et al. (1999), who demonstrated that optimal tactile performance in normally sighted subjects has an intimate relationship with the visual cortex dynamics. Interestingly, Johnson and Frostig (2016) found long, horizontal axons linking the barrel cortex to the visual cortex in rats. Even though the visual and tactile systems are closely related, we believe that the apparently earlier V1 response in comparison to that of S1 in our results may likely arise from different sized neural samples, but further work should explore possible alternative routing of tactile information to visual areas.

Functional NAs lack a strict definition, especially regarding timescales and the understanding of how interactions on the scale of single neurons relate to behavioral outcomes (Harris, 2005; Kreuz et al., 2013; Russo and Durstewitz, 2017). In this work, we used a method that characterizes NAs based on synchronous modulations in neuronal firing rate that does not support causality or time-delayed inferences. Also, it is not possible to discriminate between NAs formed by internally generated mechanisms from NAs resulting from common external inputs (Lopes-dos Santos et al., 2013). Moreover, linear PCA-based methods are fast and of high temporal resolution, but have limitations in face of the intrinsically non-linear and flexible brain dynamics (Peyrache et al., 2010; Lopes-dos Santos et al., 2013; Russo and Durstewitz, 2017). Despite the limitations due to non-stationary data, establishing the statistical threshold of assembly membership via the Marčenko-Pastur distribution considerably reduces the computational burden in comparison with surrogate methods. While this approach is much more computationally tractable than a surrogate approach, it identifies significant rate correlations which are unlikely to arise by chance, but without directly quantifying their statistical significance. Nevertheless, our findings (e.g. that NA units carry more task-related information than non-NA units) support that this approach can reveal physiologically relevant neural correlations. In this way, methods that incorporate information theory (Lopes-dos Santos et al., 2015) or statistical frameworks (Russo and Durstewitz, 2017) may enhance the assessment of distributed cortical interactions. Nevertheless, similarly to previous works (Peyrache et al., 2010; Lopes-dos Santos et al., 2011, 2013; Almeida-Filho et al., 2014; Gulati et al., 2014; Bower et al., 2015), the PCA+ICA method for characterizing NAs revealed the formation of task-related groups of neurons that are more informative about tactile discrimination than random groups of neurons, which is suggestive of functional neural interactions. Further work shall refine functional NA characterization, also considering the behavioral distinctions with which animals navigate in the scenario (Grün, 2009; Long and Carmena, 2011).

In summary, in this work we analyzed a complex dataset, recorded simultaneously from four different cortical regions in seven rats engaged in an active tactile discrimination task, which is, to the best of our knowledge, original in the literature. In addition to highlighting the neural dynamics and information content from primary sensory (S1 and V1) and associative areas (PFC and PPC), we characterize the dynamic formation of NAs, a hallmark of distributed neural interactions. Taken together, we believe our results contribute to shed light on the neural mechanisms underlying intelligent behavior.

## Author contributions

CD designed the study, performed data analysis and wrote the manuscript; AK designed the study, performed experiments and wrote the manuscript; MdS performed experiments; FL provided crucial feedback on data analysis and manuscript; RM designed the study, performed data analysis and wrote the manuscript.

### Conflict of interest statement

The authors declare that the research was conducted in the absence of any commercial or financial relationships that could be construed as a potential conflict of interest.
