# Supplementary material for: Neuronal Assemblies Evidence Distributed Interactions within a Tactile Discrimination Task in Rats
Source: Front Neural Circuits. 2018 Jan 11;11:114. doi: 10.3389/fncir.2017.00114 (PMC5768614; doi:10.3389/fncir.2017.00114)
Supplement: Supplementary file 1 [file Presentation1.PDF]

Supplementary Material: Neuronal assemblies evidence distributed interactions within a tactile discrimination task in rats

1 SUPPLEMENTARY TABLES AND FIGURES

1.1 Tables

Table S1. Population profile based on PSTH analysis: neurons were labeled either as ”Unresponsive” (last column) or ”Responsive”, based on the neuronal modulation related to the nose poke (NP). The ”responsive” cells were further classified as ”increased”, ”decreased” or ”multiphasic” activity (i.e., a combination of increased and decreased firing rate modulations). Furthermore, we characterized the magnitude modulation (i.e., the average firing rate difference within the window after NP and the baseline period), and the response duration (the average length of time for which the significant firing modulation was sustained, when compared to the baseline).

Region	Modulation	Increased	Decreased	Multiphasic	Unresponsive
PFC	Fraction	0.17 ± 0.16	0.22 ± 0.15	0.61 ± 0.24	0.11 ± 0.17
	Magnitude	2.63 ± 2.01	4.51 ± 3.79		
	Duration	0.18 ± 0.23	0.22 ± 0.61		
PPC	Fraction	0.20 ± 0.16	0.55 ± 0.21	0.25 ± 0.23	0.23 ± 0.25
	Magnitude	2.27 ± 1.58	2.41 ± 1.55		
	Duration	0.09 ± 0.03	0.15 ± 0.15		
S1	Fraction	0.22 ± 0.10	0.23 ± 0.10	0.55 ± 0.16	0.11 ± 0.15
	Magnitude	3.35 ± 2.65	2.77 ± 2.57		
	Duration	0.62 ± 1.05	0.10 ± 0.05		
V1	Fraction	0.15 ± 0.10	0.37 ± 0.32	0.48 ± 0.37	0.10 ± 0.14
	Magnitude	3.11 ± 3.20	3.37 ± 2.33		
	Duration	0.32 ± 0.63	0.17 ± 0.33		

1.2 Figures

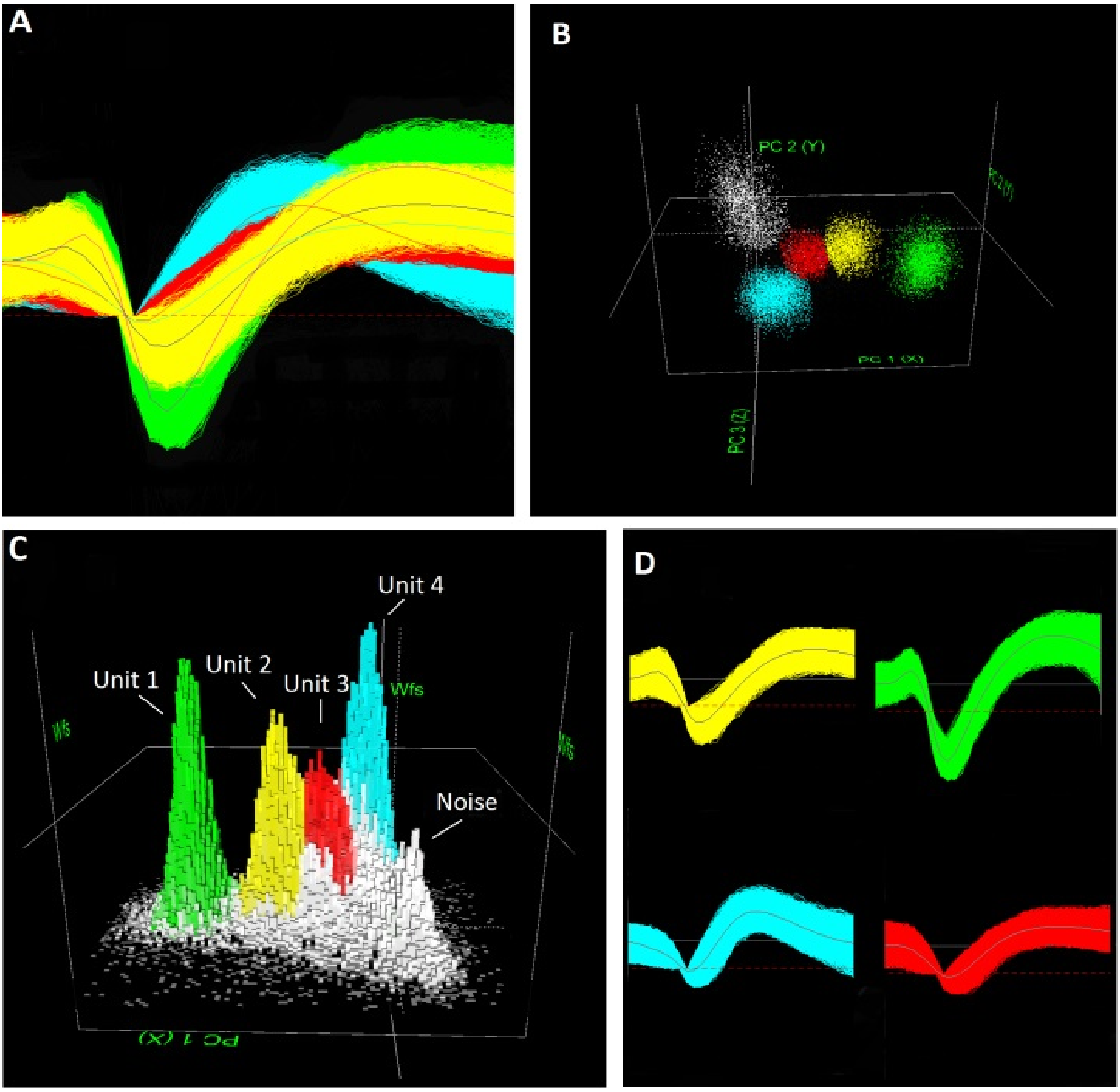

Figure S1: Spike sorting in the primary somatosensory cortex recorded during active tactile discrimination task. (A) Example of an extracellular recording from a S1 electrode. Four units were discriminated based on their different waveforms (green, yellow, red and blue). (B) Waveform cluster separation in 3D principal component space. (C) Histograms of waveform cluster for each isolated unit. (D) Examples of sorted units from the same electrode.

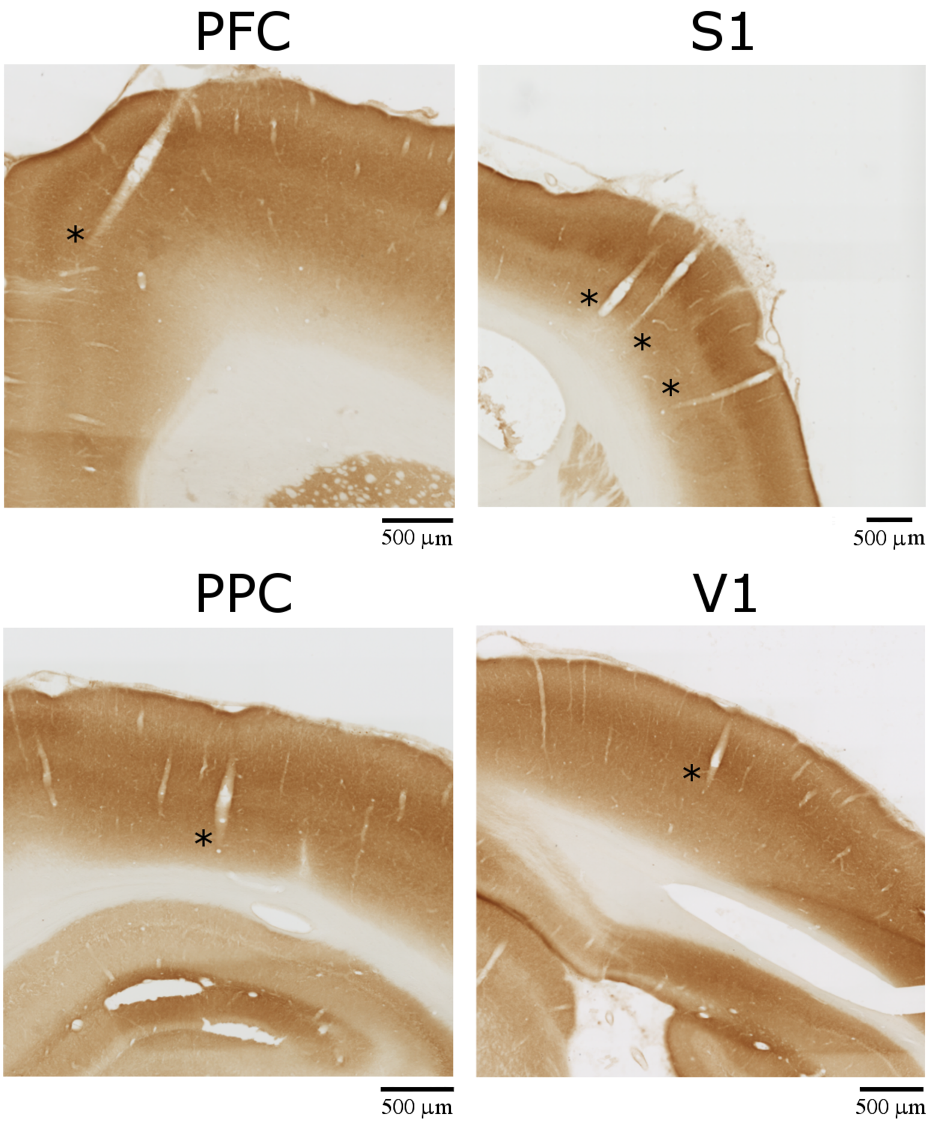

Figure S2: Histochemistry analysis of electrode traces with cytochrome-oxidase. Example of 1 electrode track in the PFC (bregma 2.16 mm), 3 electrodes in S1 (bregma -2.52 mm), 1 electrode in PPC (bregma -3.24 mm) and 1 electrode in V1 (bregma -6.24 mm). Asterisks indicate the end of electrode tracks.

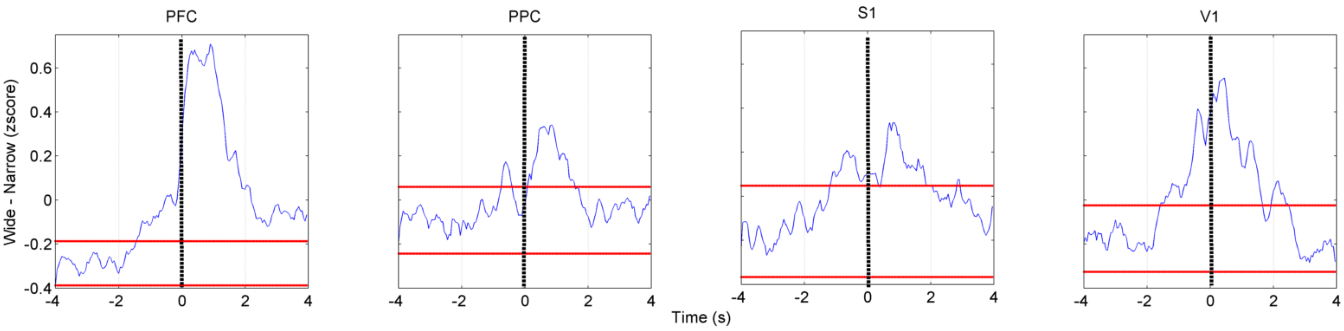

Figure S3: Mean neuronal firing rate absolute difference (z-scored) between neural responses recorded in wide and narrow scenarios. Time $t = 0s$ marks the moment rats reach the NP. Neuronal data was binned using a sliding 50 ms time-window (no overlap). Red lines mark two times the standard deviation from the average baseline ([-4 -2] s) mean firing rate.

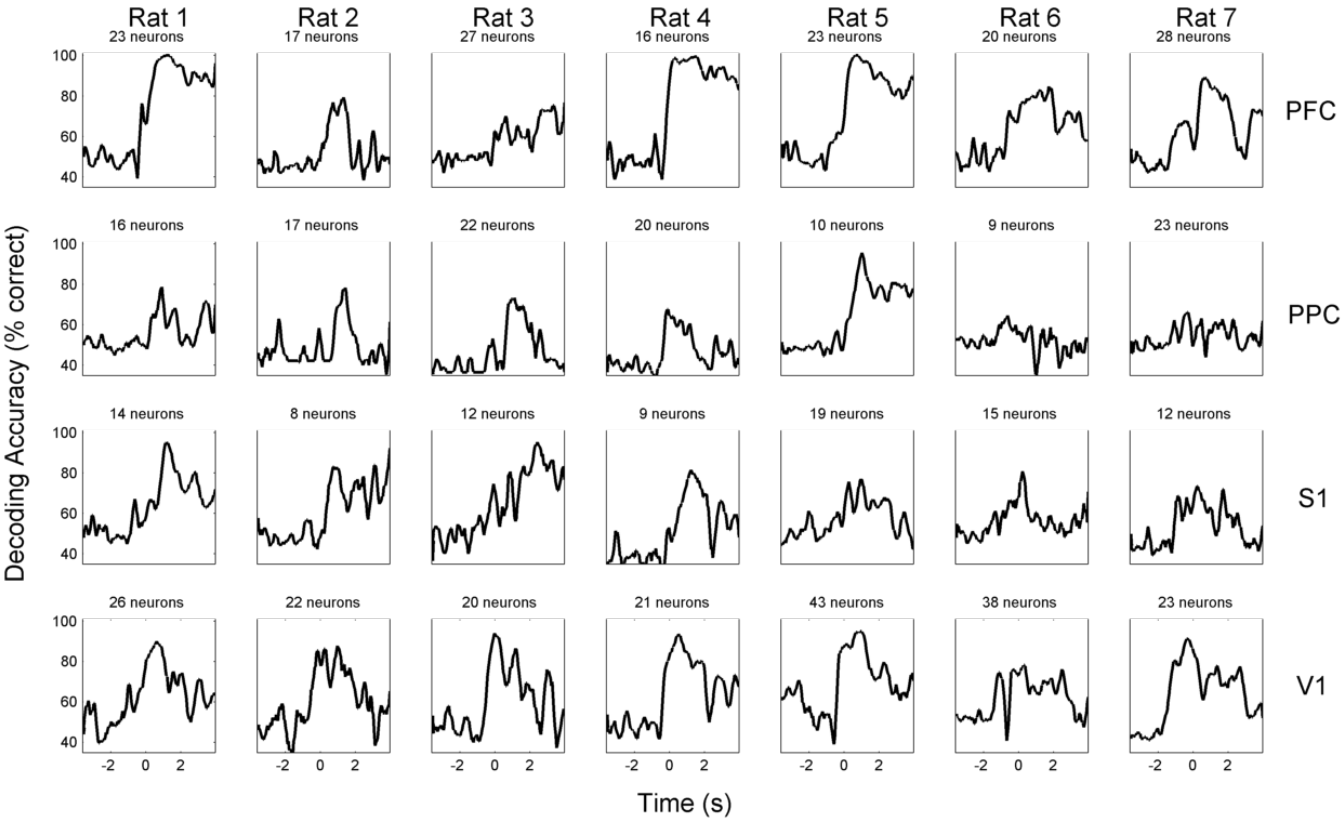

Figure S4: Mean animal response predicted with a SVM decoder, discriminated per animal and cortical region. All recorded cells within areas were employed in classification. For each trial, the decoder mapped seven consecutive 50 ms windows of spike activity (50 ms time step) into left or right reward side. Labeled time points indicate the ending of the sliding time windows. Curves are smoothed using a 5-point moving average filter. Time $t = 0s$ marks the moment rats reach the NP. Note that decoding accuracy does not vary strictly with the number of neurons.

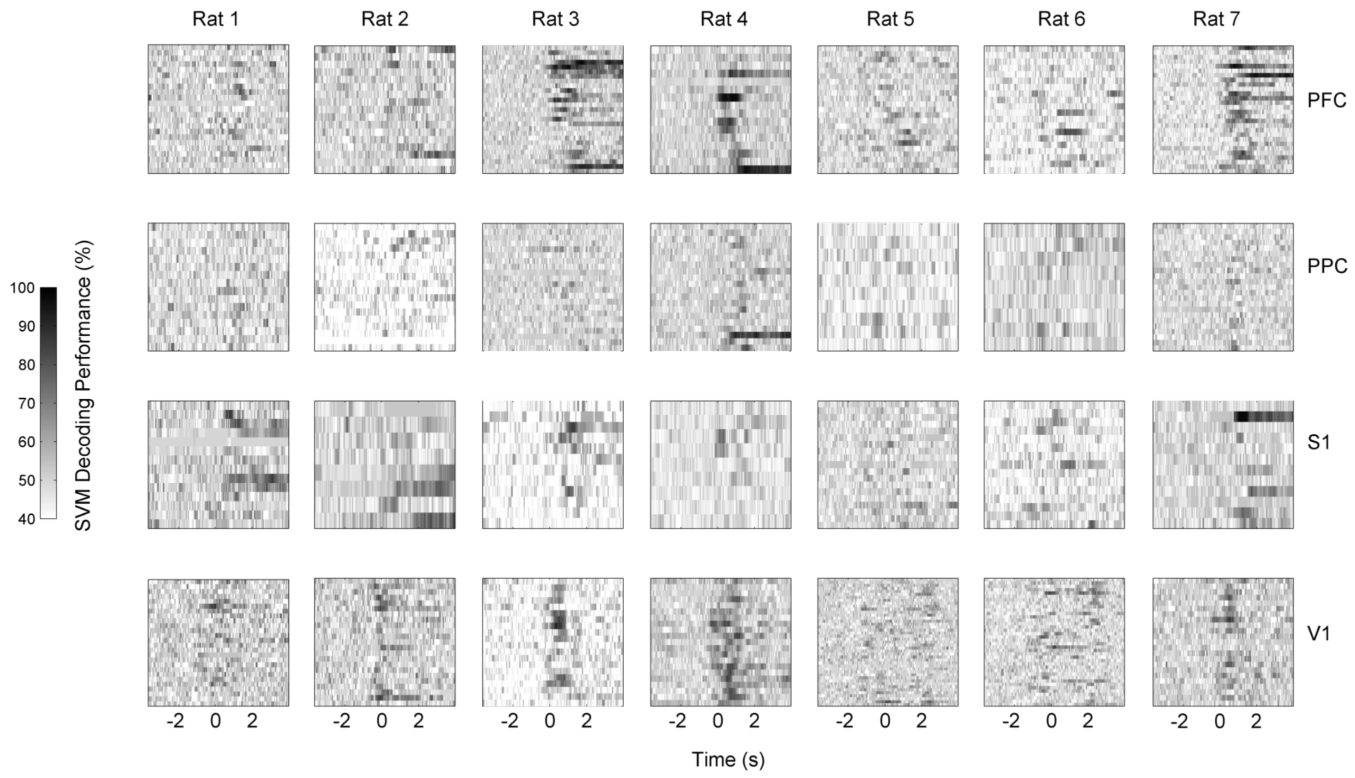

Figure S5: Single unit animal response predicted with a SVM decoder, discriminated per animal and cortical region. For each trial, the decoder mapped seven consecutive 50 ms windows of spike activity (50 ms time step) into left or right reward side. In all panels, each line refers to a single-neuron. Labeled time points indicate the ending of the sliding time windows. Time $t = 0s$ marks the moment rats reach the NP.

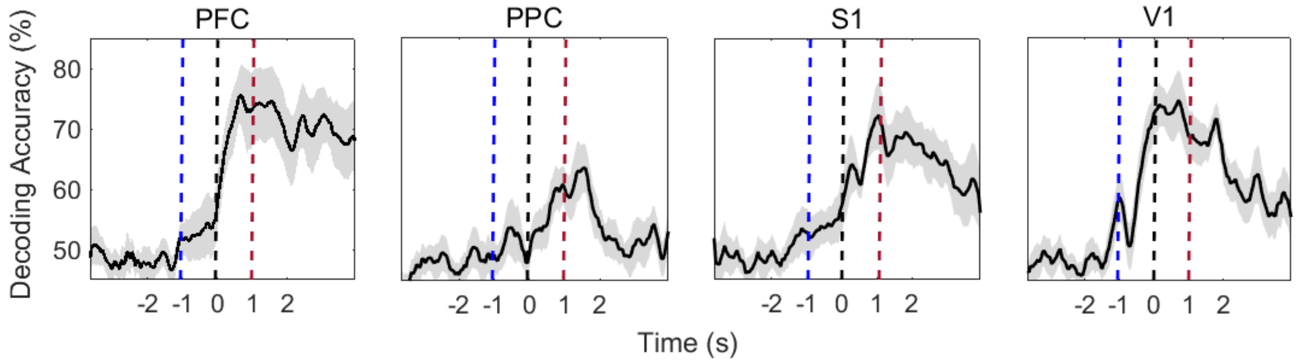

Figure S6: Mean animal response predicted with a SVM decoder. The decoder is fed with the activity from the same number of recorded cells within each cortical region. For each animal, the region with the smallest number of recorded neurons is chosen as reference, and the same number of randomly chosen neurons are considered for the remaining brain areas. For each trial, the decoder mapped seven consecutive 50 ms windows of spike activity (50 ms time step) into left or right reward side. Labeled time points indicate the ending of the sliding time windows. Curves are smoothed using a 5-point moving average filter. Time $t = 0s$ marks the moment rats reach the NP. Shaded regions relate to the standard error of the mean (calculated across the total number of animals). Dashed blue (red) vertical lines indicate the approximate beginning of trials (reward) time.

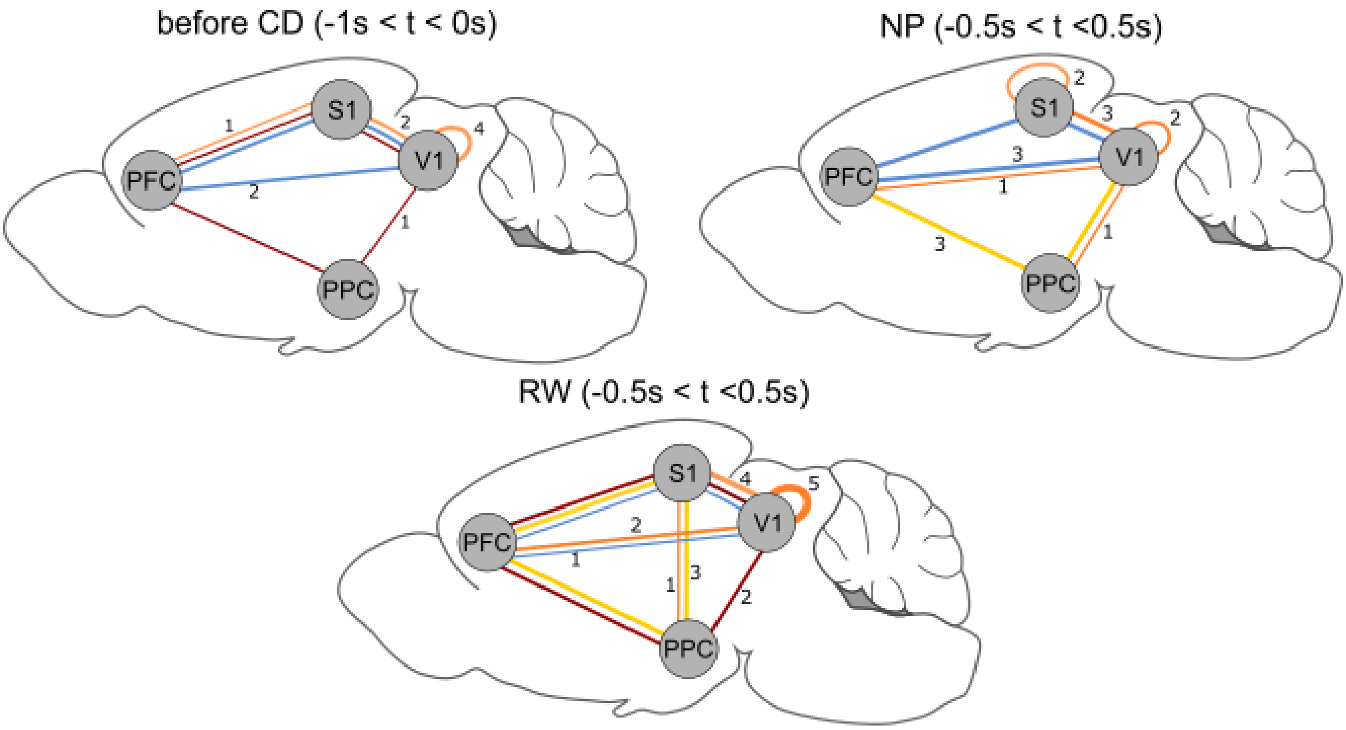

Figure S7: NA summary for all animals. Lines connect cortical areas where at least 10% of cells were engaged in the same (color-coded) NA. The thicker the connections, the greater the number of animals presenting NAs between the indicated cortical regions. The referenced events are: the aperture of the central sliding door in the behavioral apparatus (CD); the nose poke (NP); and the reward collection (RW).
